# Expression of Genes Encoding Enzymes Involved in the One Carbon Cycle in Rat Placenta is Determined by Maternal Micronutrients (Folic Acid, Vitamin B_12_) and Omega-3 Fatty Acids

**DOI:** 10.1155/2014/613078

**Published:** 2014-06-09

**Authors:** Vinita Khot, Anvita Kale, Asmita Joshi, Preeti Chavan-Gautam, Sadhana Joshi

**Affiliations:** Department of Nutritional Medicine, Interactive Research School for Health Affairs, Bharati Vidyapeeth University, Pune Satara Road, Pune 411043, India

## Abstract

We have reported that folic acid, vitamin B_12_, and omega-3 fatty acids are interlinked in the one carbon cycle and have implications for fetal programming. Our earlier studies demonstrate that an imbalance in maternal micronutrients influence long chain polyunsaturated fatty acid metabolism and global methylation in rat placenta. We hypothesize that these changes are mediated through micronutrient dependent regulation of enzymes in one carbon cycle. Pregnant dams were assigned to six dietary groups with varying folic acid and vitamin B_12_ levels. Vitamin B_12_ deficient groups were supplemented with omega-3 fatty acid. Placental mRNA levels of enzymes, levels of phospholipids, and glutathione were determined. Results suggest that maternal micronutrient imbalance (excess folic acid with vitamin B_12_ deficiency) leads to lower mRNA levels of methylene tetrahydrofolate reductase (MTHFR) and methionine synthase , but higher cystathionine b-synthase (CBS) and Phosphatidylethanolamine-N-methyltransferase (PEMT) as compared to control. Omega-3 supplementation normalized CBS and MTHFR mRNA levels. Increased placental phosphatidylethanolamine (PE), phosphatidylcholine (PC), in the same group was also observed. Our data suggests that adverse effects of a maternal micronutrient imbalanced diet may be due to differential regulation of key genes encoding enzymes in one carbon cycle and omega-3 supplementation may ameliorate most of these changes.

## 1. Introduction

**Figure 1:**
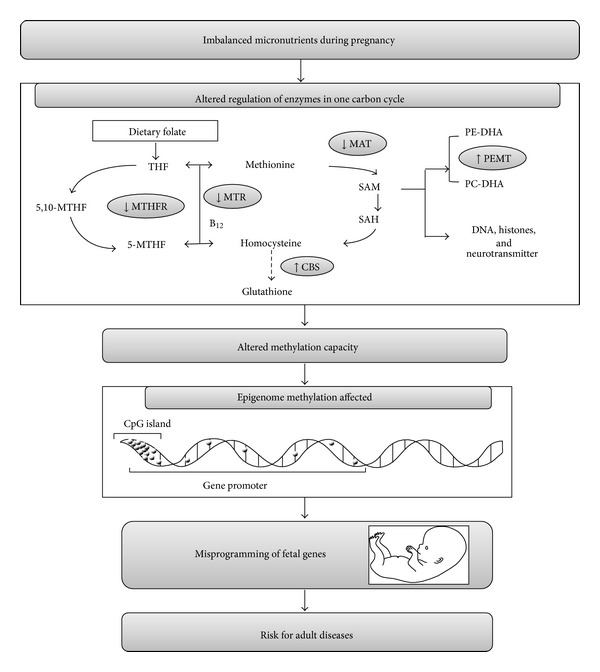
Role of altered micronutrients and omega-3 in the epigenetic regulation of developing fetus. The key metabolic components: THF: tetrahydrofolate, 5,10-MTHF: 5,10-methylene tetrahydrofolate; 5-MTHF: 5-methylene tetrahydrofolate; B_12_: vitamin B_12_; methionine; SAM: S-adenosyl methionine; SAH: S-adenosyl homocysteine; homocysteine; glutathione; PE-DHA: phosphatidylethanolamine with docosahexaenoic acid attached to position 2; PC-DHA: phosphatidylcholine with docosahexaenoic acid attached to position 2; DNA: deoxyribonucleic acid, histone. Key enzymes: MTHFR: methylene tetrahydrofolate reductase; MTR: methionine synthase; PEMT: phosphatidylethanolamine methyl transferase; CBS: cystathionine beta Synthase.

Micronutrients, especially folic acid and vitamin B_12_, although required in tracing quantities, play a major role in pregnancy and fetal outcome and have implications in the regulation of various metabolic processes in the body [1]. These micronutrients play a key role in the one carbon metabolism for methionine and S-adenosylmethionine (SAM) synthesis and homocysteine (Hcy) clearance [2, 3]. Briefly, the enzyme methylene tetrahydrofolate reductase (MTHFR) converts dietary folates to 5 methyl tetrahydrofolate (5-MTHF), which donates the methyl group for the remethylation of homocysteine to methionine catalyzed by methionine synthase (MS/MTR). Methionine adenosyl transferase (MAT) acts on this methionine to produce SAM. On donating the methyl group to most methyl acceptors, SAM is converted to s-adenosyl homocysteine (SAH) and further hydrolysed to Hcy, which is then either remethylated to methionine or is utilized for cysteine synthesis catalyzed by cystathionine b-synthase (CBS) (Figure 1).

Phospholipids, neurotransmitters, and deoxyribonucleic acid (DNA) are the major methyl acceptors. Phospholipids utilize almost 14% of the methyl groups produced for the conversion of phosphatidylethanolamine (PE) to phosphatidylcholine (PC), [4, 5] in a reaction catalyzed by phosphatidylethanolamine-N-methyltransferase (PEMT) [6, 7]. The availability of folate and vitamin B_12_ may therefore alter the methylation of PE to PC, further affecting DHA concentrations in plasma and tissues (mainly brain) [2, 8]. Lower conversion of PE to PC will lead to excess methyl group availability for other transmethylation reactions such as DNA methylation and may lead to altered chromatin remodeling and gene expression (Figure 1). Thus, the metabolisms of folic acid, vitamin B_12_, and DHA are interdependent on each other [2] possibly through the one carbon methyl cycle.

Studies on cell culture have reported that DHA decreases plasma homocysteine by regulating enzyme activity and mRNA expression involved in methionine metabolism [9]. Similarly the effect of n3 PUFA's on these critical enzymes of the homocysteine metabolism has been documented [10]. But to date no studies have shown the effect of DHA supplementation to a maternal diet imbalanced in micronutrients on the mRNA levels of the enzyme genes involved in the homocysteine metabolism.

The rationale behind the present study design was that, in developing countries, like India, micronutrient deficiencies are common and are associated with poor pregnancy outcomes [11]. In view of this, folic acid supplementation is undertaken during pregnancy as per the National Prophylaxis Program to improve birth weights and to prevent incidences of neural tube defects. Further, most Indian diets are deficient in vitamin B_12_ due to vegetarianism which may lead to an imbalance in the vital micronutrients like folic acid and vitamin B_12_.

We have recently reported that an imbalance in maternal micronutrients (folic acid, vitamin B_12_) in Wistar rats increases maternal oxidative stress, decreases placental and pup brain DHA levels, and decreases placental global methylation levels [12, 13]. Based on these studies, we now hypothesize that an imbalance in the levels of maternal micronutrients (folic acid, vitamin B_12_) during pregnancy will differentially program the expression of genes encoding critical enzymes (expressed by mRNA levels) involved in the one carbon cycle (described earlier in Figure 1), and omega-3 fatty acid supplementation may prevent some of these changes.

## 2. Materials and Methods

All the experimental procedures were in accordance with CPCSEA guidelines (Committee for the Purpose of Control and Supervision of Experimental Animals), Govt. of India and were approved by the Bharati Vidyapeeth Institutional Animal Ethics Committee (IAEC) (IAEC/CPCSEA/2618). This institute is recognized to undertake experiments on animals in accordance with CPCSEA guidelines.

### 2.1. Animals

**Figure 2:**
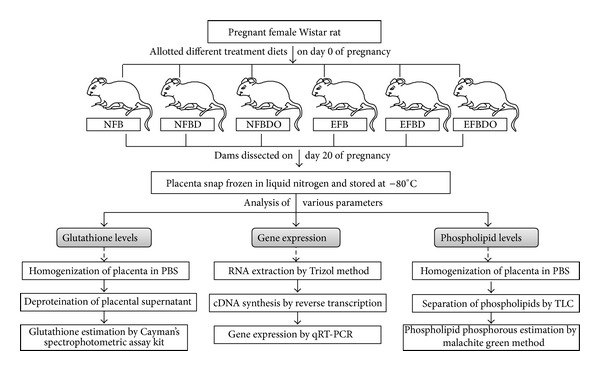
Study Design. NFB: normal folic acid, normal vitamin B_12_; NFBD: normal folic acid, vitamin B_12_ deficient; EFB: excess folic acid, normal vitamin B_12_; EFBD: excess folic acid, vitamin B_12_ deficient; NFBDO: normal folic acid, vitamin B_12_ deficient, omega-3 supplemented EFBDO: excess folic acid, vitamin B_12_ deficient, omega-3 supplemented.

The detailed description of the animal protocol has been published by us earlier [12–15]. The animals were bred at 3 months of age, till which they were fed control diet. After confirmation of pregnancy, these pregnant female rats were allocated randomly to the following six (control and five experimental) diets (eight on each). All dams were delivered by C section on day 20 of gestation. Placental tissues were snap-frozen in liquid nitrogen and stored at −80°C until further analysis (Figure 2).

### 2.2. Diet

Diet composition was as per AIN 93G guidelines and has been described by us earlier [12–14]. Protein level in the control and treatment diets was 18%. Vitamin-free casein was used for all treatment diets. The diet is prepared manually in our laboratory by mixing all the various components recommended. The fish oil capsules were weighed and added during this preparation only to the omega supplemented diets and not to control.

The pregnant dams were randomly divided into six dietary groups (control and five experimental). The six groups, with each containing eight dams, are equally divided at two folic acid levels (i.e., 2 mg and 8 mg folic acid/kg diet) with the presence and absence of vitamin B_12_. In addition, omega-3 (DHA, 120 mg and EPA, 180 mg, source: Merck Maxepa capsules) supplementation to the vitamin B_12_ deficient groups was undertaken (Table 1). The three at normal folic acid levels (2 mg/kg diet) are normal folic acid and vitamin B_12_ (NFB/Control), normal folic acid and vitamin B_12_ deficient (NFBD), and omega-3 supplementation at normal folic acid and vitamin B_12_ deficient levels (NFBDO). The other three groups at supplemented folic acid levels (8 mg/kg diet) are excess folic acid and vitamin B_12_ (EFB), excess folic acid and vitamin B_12_ deficient (EFBD), and omega-3 supplementation at excess folic acid and vitamin B_12_ deficient levels (EFBDO). The detailed fatty acid composition of all the diets has been reported by us earlier [14]. Briefly, the EPA and DHA content in various diets (g/100 g fatty acids) is as follows: EPA (NFB = 0; NFBD = 0; EFB = 0; EFBD = 0; NFBDO = 5.64; EFBDO = 5.62) and DHA (NFB = 0; NFBD = 0; EFB = 0; EFBD = 0; NFBDO = 3.15; EFBDO = 3.13).

### 2.3. Placental mRNA Levels

Total RNA from placental samples was isolated using Trizol method and quantified by using the Eppendorf biophotometer machine. One microgram of total RNA was transcribed to cDNA with the High-Capacity cDNA reverse transcription Kit (Applied Biosystems, Foster City, CA, USA). Real-time quantitative PCR for the enzymes* MTHFR*,* MTR*,* MAT2a*,* CBS*,* PEMT,* and Glyceraldehyde-3-phosphate dehydrogenase (*GAPDH*) was performed using the Applied Biosystems 7500 system. The mRNA level of the gene of interest was computed with respect to* GAPDH* mRNA to normalize for variation in the quality of RNA and the amount of input cDNA. Real-time PCR was performed with the TaqMan Universal PCR Master Mix (Applied Biosystems, USA) using cDNA equivalent to 100 ng total RNA. Ct-values were set in the exponential range of the amplification plots using the 7500 System Sequence Detection Software v2.0.5. ΔCt-values corresponded to the difference between the Ct-values of the genes examined and those of the* GAPDH* (internal control) gene [16, 17]. The mRNA levels of genes were calculated and expressed as 2^ΔCt^. The following FAM dye-labeled TaqMan assays (Applied Biosystems, USA) were used in this study:* GAPDH* (Rn99999916_s1);* MTHFR* (Rn01515583_m1);* MTR* (Rn00578368_m1);* MAT2a* (Rn01643368_g1);* PEMT* (Rn00564517_m1);* CBS* (Rn00560948_m1).

### 2.4. Placental Glutathione Levels

The placental samples were weighed, homogenized (PBS buffer), and centrifuged at 4°C and 10,000 rpm for 10 minutes. The supernatant was deproteinized (using MPA reagent) prior to analysis. The supernatant was also used for protein analysis by Lowry method. Glutathione levels were estimated from dam placenta supernatant using Cayman's Glutathione Assay kit (Catalog number 703002). The kit utilizes glutathione reductase for measurement of both GSH and GSSG and the assay reflects total glutathione. The samples were stored at −80°C until processing. With the glutathione moiety being unstable, care was taken that the samples were stored on ice after deproteination and assayed within 20 minutes after processing.

Briefly, the sulfhydryl group of glutathione (GSH) reacts with 5,5′-dithio-bis-2-(nitro benzoic acid) (DNTB/Ellman's reagent) to produce a yellow colored 5-thio-2-nitrobenzoic acid (TNB). The rate of production of TNB is directly proportional to the GSH in the sample. The kit uses GSSH, the disulfide dimer of GSH, as standard. Placental glutathione concentration is expressed as *μ*M GSH/mg protein of the sample.

### 2.5. Phospholipid Analysis of Placenta

#### 2.5.1. Homogenization of Placenta

The placental samples were weighed, homogenized (PBS buffer), and centrifuged at 4°C and 10,000 rpm for 10 minutes. The supernatant was used for protein analysis by Lowry method. The pellet was resuspended in 0.5 mL of the buffer and processed further.

#### 2.5.2. Separation of Phospholipids by Thin Layer Chromatography (TLC)

The phospholipids, phosphatidylethanolamine (PE) and phosphatidylcholine (PC), were extracted, separated, and quantified using a modified method of Folch et al. [18, 19]. The resuspended sample was treated with a chloroform/methanol mixture (2 : 1, by vol.) (twenty times the sample volume) and incubated in the dark for one hour. The mixture was filtered and the filtrate was dried by argon. The dried sample was resuspended in 300 *μ*L of chloroform: methanol mixture (2 : 1), sonicated (15 minutes, cold water) and then spotted on the TLC plate (silica gel 60 aluminum sheets obtained from Merck). The plate was developed in chloroform/methanol/water, (12 : 5 : 0.9, by vol.) for 20 minutes. After thorough drying, the plate was exposed to iodine fumes to identify the phosphatidylethanolamine (PE) and phosphatidylcholine (PC) spots in comparison to standards procured from Sigma chemicals. The PE and PC spots were scraped into a conical tube for phosphorous estimation.

#### 2.5.3. Phospholipid Phosphorous Estimation

The scraped PE and PC spots were digested in perchloric acid (overnight at 100°C). The PE and PC phospholipid fractions were estimated quantitatively by using the malachite green colorimetric method for phosphorous estimation [20]. Dihydrogen potassium phosphorus (500 pmoles–3000 pmoles) was used as phosphorous standard. The phospholipid concentrations were calculated on molar basis by multiplying with 25 [21, 22], and values were expressed as mg phospholipids/gram of placental tissue. The PC : PE ratio indicative of the PE to PC conversion is represented.

### 2.6. Statistical Analysis

The data were analyzed using SPSS/PC+ package (Version 20.0, Chicago, IL). Values are expressed as mean ± SD. The statistical analysis was carried out on placentae of eight dams per group. The placental tissue of each dam in each group was used for the various biochemical and molecular estimations. The actual number of animals per group is indicated for each of the parameters analyzed in the respective table and figures. The treatment groups were compared with the control group by ANOVA and the post hoc least significant difference test.

## 3. Results

### 3.1. Placental One Carbon Cycle Enzyme mRNA Levels

All the enzyme mRNA levels were detected in the placenta and those are presented in Figure 2.

#### 3.1.1. MTHFR Levels

**Figure 3:**
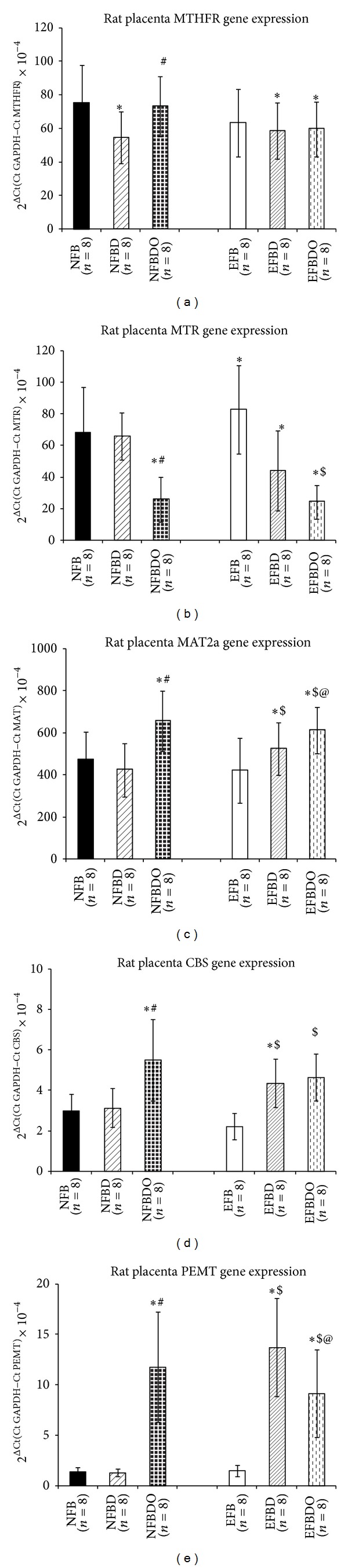
Rat placental mRNA levels of key enzyme genes in the one carbon cycle. (a) MTHFR levels; (b) MTR levels; (c) MAT2a levels; (d) CBS levels; (e) PEMT levels. Values are expressed as Mean ± SD (*n* = 8 for all). Significance: *P < 0.01 when compared to control (normal folate, normal B_12_); ^#^ *P* < 0.01 when compared to NFBD (normal folate, B_12_ deficient); ^$^ *P* < 0.01 when compared to EFB (excess folate, normal B_12_), ^@^ *P* < 0.01 when compared to EFBD (excess folate, B_12_ deficient); methylene tetrahydrofolate reductase (MTHFR), methionine tetrahydrofolate reductase (MTR), methionine adenosyl transferase (MAT), cystathionine b-synthase (CBS), and Phosphatidylethanolamine-N-methyltransferase (PEMT). NFB: normal folic acid, normal vitamin B_12_; NFBD: normal folic acid, vitamin B_12_ deficient; EFB: excess folic acid, normal vitamin B_12_; EFBD: excess folic acid, vitamin B_12_ deficient; NFBDO: normal folic acid, vitamin B_12_ deficient, omega-3 supplemented EFBDO: excess folic acid, vitamin B_12_ deficient, omega-3 supplemented.

The* MTHFR* mRNA levels were lower (*P* < 0.01) in both the vitamin B_12_ deficient groups at normal and excess levels of folic acid (NFBD and EFBD) as compared to the control. Omega-3 fatty acid supplementation at the normal folic acid level group (NFBDO) led to higher (*P* < 0.01) mRNA levels as compared to NFBD. However, omega supplementation did not change the mRNA levels at excess folic acid levels with vitamin B_12_ deficiency (EFBDO) (Figure 3(a)).

#### 3.1.2. MTR Levels

The MTR enzyme mRNA levels were comparable to control in the vitamin B_12_ deficient group at normal folic acid level (NFBD). The* MTR* mRNA levels were higher (*P* < 0.01) in the normal vitamin B_12_ with excess folic acid group (EFB) as compared to control. On the other hand, vitamin B_12_ deficiency in the presence of excess folic acid levels (EFBD) resulted in lower (*P* < 0.01 for both) mRNA levels of the enzyme as compared to control and EFB. Omega-3 fatty acid supplementation at both the folic acid levels (NFBDO and EFBDO) showed a decrease (*P* < 0.01 for all) in the mRNA levels as compared to the control and both the respective vitamin B_12_ deficient groups (Figure 3(b)).

#### 3.1.3. MAT2a Levels

The MAT2a mRNA levels were comparable to control in the vitamin B_12_ deficiency in the presence of normal folic acid levels (NFBD). However, an increase (*P* < 0.01 for both) in the* MAT2a *mRNA levels was found in the vitamin B_12_ deficient group in the presence of excess folic acid (EFBD) as compared to control and EFB. Omega-3 fatty acid supplementation at the both folic acid levels (NFBDO and EFBDO) showed higher (*P* < 0.01 for both) mRNA levels as compared to the control and respective vitamin B_12_ deficient groups (NFBD and EFBD) (Figure 3(c)).

#### 3.1.4. CBS Levels

The CBS mRNA levels in the vitamin B_12_ deficient group at normal folic acid level (NFBD) were comparable to control. However, excess folic acid with a vitamin B_12_ deficiency (EFBD) resulted in higher (*P* < 0.01 for all)* CBS* mRNA levels as compared to the control, NFBD, and EFB groups. Omega-3 fatty acid supplementation at both normal and excess folic acid levels (NFBDO and EFBDO) showed higher (*P* < 0.01 for all) mRNA levels as compared to the control and respective vitamin B_12_ deficient groups (NFBD and EFBD) (Figure 3(d)).

#### 3.1.5. PEMT Levels

The PEMT mRNA enzyme levels were comparable to control in NFBD and EFB groups. The PEMT mRNA levels in vitamin B_12_ deficiency in presence of excess folic acid levels (EFBD) were higher (*P* < 0.01 for all) as compared to control, NFBD, and EFB. Omega-3 fatty acid supplementation at the normal folic acid level (NFBDO) led to higher (*P* < 0.01 for both) mRNA levels as compared to the control and NFBD. However, omega-3 fatty acid supplementation at excess folic acid levels (EFBDO) showed higher (*P* < 0.01) mRNA levels as compared to control but lower (*P* < 0.05) as compared to the EFBD group (Figure 3(e)).

### 3.2. Placental Glutathione Levels

**Figure 4:**
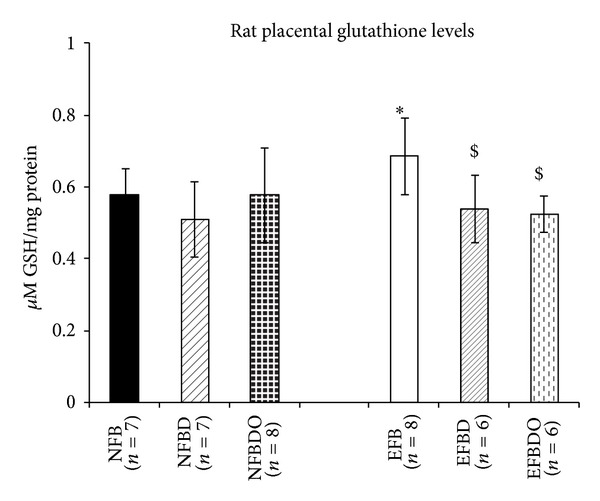
Rat placental glutathione levels. Values are expressed as Mean ± SD. Significance: *P < 0.05 when compared to control (normal folate, normal B_12_); ^$^ *P* < 0.05 when compared to EFB (excess folate, normal B_12_). NFB: normal folic acid, normal vitamin B_12_; NFBD: normal folic acid, vitamin B_12_ deficient; EFB: excess folic acid, normal vitamin B_12_; EFBD: excess folic acid, vitamin B_12_ deficient; NFBDO: normal folic acid, vitamin B_12_ deficient, omega-3 supplemented EFBDO: excess folic acid, vitamin B_12_ deficient, omega-3 supplemented.

The glutathione level in the maternal placenta of NFBD group was comparable to control. These levels increased (*P* < 0.01) in the maternal group with normal vitamin B_12_ in the presence of excess folic acid (EFB) as compared to control. However, decreased (*P* < 0.01) glutathione levels were found in the vitamin B_12_ deficient group in the presence of excess folic acid levels (EFBD) as compared to EFB group. Omega-3 fatty acid supplementation at normal folic acid levels (NFBDO) resulted in glutathione levels comparable to control, but this was not observed in the EFBDO group (Figure 4).

### 3.3. Phospholipid Estimations

#### 3.3.1. Levels of PE

The PE fraction of the placental membrane phospholipid was higher (*P* < 0.01) in both the vitamin B_12_ deficient groups (NFBD and EFBD) as compared to the control. The PE levels in the omega-3 fatty acid supplemented group at normal folic acid levels (NFBDO) were comparable to NFBD group. In contrast, on omega-3 fatty acid supplementation at the excess folic acid levels (EFBDO) showed an increase (*P* < 0.05 for both) as compared to control and EFBD groups (Table 2).

#### 3.3.2. Levels of PC

The PC fraction of the placental membrane phospholipid was also higher (*P* < 0.01 for both) in both the vitamin B_12_ deficient groups (NFBD and EFBD) as compared to the control. Omega-3 fatty acid supplementation at the normal folic acid levels (NFBDO) showed an increase (*P* < 0.05) in the PC concentration as compared to control. Omega-3 fatty acid supplementation at the excess folic acid levels (EFBDO) showed a decrease (*P* < 0.05) in the PC concentration as compared to EFBD (Table 2).

#### 3.3.3. PC : PE Ratio

In contrast to NFBD, PC : PE ratio increased (*P* < 0.05) in the EFBD group as compared to control. Omega-3 supplementation at the excess folic acid levels (EFBDO) showed a decrease (*P* < 0.05) in the ratio as compared to EFBD (Table 2).

## 4. Discussion

This study for the first time demonstrates several interesting findings in relation to maternal key micronutrients (folic acid, vitamin B_12_) and omega-3 fatty acids on the placental mRNA levels of key genes encoding enzymes of the one carbon cycle, phospholipids PE and PC and glutathione levels.

The findings are as follows. (1) Vitamin B_12_ deficiency at normal levels of folic acid (NFBD) showed lower mRNA levels only for gene encoding* MTHFR* enzyme. However the supplementation of omega-3 fatty acids (NFBDO) normalized the mRNA levels of* MTHFR*, reduced the* MTR* mRNA levels, but increased the levels of* MAT2a*,* PEMT,* and* CBS*. (2) Vitamin B_12_ deficiency in the presence of excess folic acid levels (EFBD) resulted in lower mRNA levels of* MTR* (decreased), and higher* PEMT* and* CBS *mRNAlevels. Supplementation of omega-3fatty acids to this group increased the levels of* MAT2a* and decreased the* MTR* and* PEMT *mRNA levels. (3) The phospholipid fractions (particularly PE and PC) were higher in both the vitamin B_12_ deficient groups (NFBD and EFBD) compared to control while omega-3 fatty acid supplementation reduced both levels. Thus our results strongly indicate that a maternal diet with imbalances in micronutrients, particularly vitamin B_12_ deficiency in the presence of excess folic acid levels, resulted in the most adverse effects on expression of key genes encoding enzymes in one carbon cycle (Figure 1).

The present study was carried out to examine the mechanistic aspects of omega 3 fatty acid supplementation keeping in mind the omega 3 : omega 6 ratio of 1 : 1, as it has been established that this ratio is ideal for human diet [23, 24]. In India the current omega 3 : omega 6 ratio is reported to be around 1 : 26 in pregnant women, which is much higher than the recommended ratio of 1 : 5–10 for optimal health benefit [25]. The Indian population being mainly vegetarian, the source of omega fatty acids is the vegetable oil consumed in the diet. The oils used like safflower oil, sunflower oil, and so forth are rich in LA [25], which disturbs the required omega 3 : omega 6 ratio of 1 : 1.

In the group with vitamin B_12_ deficiency at normal folate (NFBD) there was a reduction in mRNA levels of* MTHFR*. The diet altered in folic acid and vitamin B_12_ may lead to low influx of methyl groups for the remethylation of the homocysteine [26]. This lowered remethylation may be due to the enzyme* MTHFR* [27, 28], which is reflected in the observed lower mRNA levels.

Omega-3 fatty acid supplementation to this group (i.e., NFBDO) was able to normalize the* MTHFR* but decreased the* MTR* and increased the levels of* MAT2a*,* PEMT*, and* CBS *mRNA levels. In a recent study, omega-3 fatty acids in HepG-2 control cells have been shown to upregulate the* MTHFR* enzyme expression but had no effect on the* MTR* expression [10]. Our earlier studies in the same altered micronutrient groups (NFBD and NFBDO) have reported that vitamin B_12_ deficiency reduces the levels of DHA in plasma and placenta [12, 13]. These enzyme alterations may reflect the compensatory changes occurring in the placenta as a result of increased levels of PE and PC and effective conversion of PE to PC (higher PC : PE ratio) activated by the lowered DHA levels in the plasma.

In the group with vitamin B_12_ deficiency in the presence of excess folic acid levels (condition for “methyl trap”), there was a reduction of* MTHFR* and* MTR* mRNA levels but increase in levels of* MAT2a*,* PEMT,* and* CBS *mRNA levels. The decrease in the* MTHFR* and the* MTR* mRNA levels obtained in this group as compared to control may signify the altered remethylation of homocysteine in the placenta. However, our earlier study has reported no significant difference in homocysteine levels in the same diet groups in rats [13]. This indicates that homocysteine may be eliminated through the alternate transsulfuration pathway which is supported by the increased* CBS* mRNA levels. Furthermore, along with the higher* PEMT *mRNAlevels, both PE and PC phospholipids were also very high with resultant high ratio of PC : PE. This again indicates a possible compensatory mechanism for the transport of DHA in plasma through the one carbon cycle. However, it will be important to confirm these findings directly using labeled carbon assays in future studies.

Supplementation of omega-3 fatty acids at excess folic acid levels did not normalize the levels of* MTHFR*, reduced the levels of* MTR*, and again increased the levels of* MAT2a*,* PEMT* (though less than EFBD group), and* CBS*. The levels of PE and PC were higher than EFB group but were lower than that in EFBD group with similar changes in the PC : PE ratio. We have earlier reported an increase in DHA levels in the placenta and plasma as a consequence of omega-3 fatty acid supplementation [12, 13] which may suppress the* PEMT* levels. The reduced PC : PE ratio in the presence of omega-3 fatty acids also supports this decrease in the* PEMT* mRNA levels.

There are no other studies which have systematically examined these enzyme changes under these dietary issues and hence the implications of these findings may not currently be very clear. However, earlier studies have well established that SAM donates three methyl groups for the conversion of PE-DHA to PC-DHA by the* PEMT* enzyme [4, 8], and the* PEMT* pathway is also the major contributor for the availability and transport of PUFAs like DHA to other tissues [6, 7]. The* PEMT* enzyme activity to date has been principally studied in the liver [29, 30]. Other studies also report the* PEMT* gene expression in tissues like brain [5, 31] and adipose tissue [32]. The present study for the first time explores the hypothesis that the* PEMT* gene is expressed in the placenta for the transport of DHA to the fetus. It is also possible that the* PEMT* gene may be hypomethylated. However, gene-specific methylation studies are required as it is known that DNA methylation plays a role in mediating the expression of most genes in the cell [33]. This may be partly supported by the lower global methylation patterns in the placenta in the micronutrient imbalanced group which we have reported earlier [13]. The present study has only assessed effects on mRNA expression, and it will be important to establish whether these translate into changes in protein levels and activities of these enzymes. In addition, assessing the separate contributions of the 2 major n-3 LCPUFA, EPA, and DHA, and how they compare to the effects of n-6 PUFA, will provide greater insights into the role of these individual fatty acids in regulating placental one-carbon metabolism and will be kept in mind while designing future studies.

Our results for the first time suggest that the adverse effects of a maternal micronutrient (folic acid and vitamin B_12_) imbalance diet as indicated by the altered major phospholipids and the levels of glutathione and mRNA levels of the key genes encoding enzymes of the one carbon cycle in the rat placenta may also be determined by the levels of other components such as DHA containing phospholipids. These changes may also have important implications for the epigenetic programming of the developing fetus (Figure 1).

## Figures and Tables

**Table 1:** Composition of the diets.

S No	Diets	NFB (g/kg)	NFBD (g/kg)	NFBDO (g/kg)	EFB (g/kg)	EFBD (g/kg)	EFBDO (g/kg)
1	Corn starch	398	398	398	398	398	398
2	Casein	200	200	200	200	200	200
3	Dextrinized starch	132	132	132	132	132	132
4	Sucrose	100	100	100	100	100	100
5	Soya bean oil	70	70	25	70	70	25
6	Fish oil [DHA, EPA]^a^	0	0	45	0	0	45
7	Fiber	50	50	50	50	50	50
8	Mineral mixture^b^	35	35	35	35	35	35
9	Vitamin mixture^c^	10	10	10	10	10	10
	Folic acid	0.002	0.002	0.002	0.008	0.008	0.008
	Vitamin B_12_	0.025	0	0	0.025	0	0
10	Cystine	3	3	3	3	3	3
11	Choline bitartrate	2.5	2.5	2.5	2.5	2.5	2.5
12	Tertiary butyl	0.014	0.014	0.014	0.014	0.014	0.014
13	Total energy (kJ)	15.7	15.7	15.7	15.7	15.7	15.7

^a^The diet was supplemented with Maxepa capsules (Merck) which contains a combination of DHA (120 mg) and EPA (180 mg) per capsule. The source of the DHA and EPA is fish lipid oil in the form of free fatty acids.

^b^Mineral mixture (g/kg mixture): calcium carbonate, 357; potassium phosphate, 196; potassium citrate, 70.78; sodium chloride, 78; potassium sulphate, 46.6; magnesium oxide, 24; ferric citrate, 6.06; zinc carbonate, 1.65; manganous carbonate, 0.63; cupric carbonate, 0.3; potassium iodate, 0.01; sodium selenate, 0.01; ammonium paramolybdate, 0.007; sodium metasilicate, 1.45; chromium potassium sulphate, 0.275; lithium chloride, 0.01; boric acid, 0.08; sodium fluoride, 0.06; nickel carbonate, 0.03; ammonium vanadate, 0.006; sucrose, 221.02.

^c^Vitamin mixture (g/kg mixture) of nicotinic acid, 3; calcium pantothenate, 1.6; pyridoxine-HCl, 0.7; thiamin-HCl, 0.6; riboflavin, 0.6; D-biotin, 0.02; vitamin B_12_ (in 0.1% Mannitol), 2.5; vitamin E, 15; vitamin A, 0.8; vitamin D-3, 0.25; vitamin K, 0.075; folic acid, 0.2 (control), and sucrose 974.655, was used to make total weight of the vitamin mixture to 1 kg.

NFB: normal folic acid, normal vitamin B_12_; NFBD: normal folic acid, vitamin B_12_ deficient; EFB: excess folic acid, normal vitamin B_12_; EFBD: excess folic acid, vitamin B_12_ deficient; NFBDO: normal folic acid, vitamin B_12_ deficient, omega-3 supplemented EFBDO: excess folic acid, vitamin B_12_ deficient, omega-3 supplemented.

**Table 2:** Dam placenta phospholipid levels in different groups (Mean ± SD).

Phospholipid (mg/g tissue)	NFB (*n* = 8)	NFBD (*n* = 8)	NFBDO (*n* = 8)	EFB (*n* = 8)	EFBD (*n* = 8)	EFBDO (*n* = 8)
PE levels	5.3 ± 1.6	23.5 ± 10.7**	11.9 ± 5.7	4.9 ± 2.2	21.5 ± 9.07^∗$^	10.6 ± 3.8^∗$^
PC levels	7.7 ± 2.6	16.8 ± 8.5**	10.3 ± 4.1*	5.8 ± 1.5	34.8 ± 7.1^∗$^	9.3 ± 4.7^@^
PC : PE ratio	1.06 ± 0.53	0.72 ± 0.35	0.94 ± 0.34	1.16 ± 0.47	1.81 ± 0.81^∗#^	0.83 ± 0.24^@^

**P < 0.01 compared to NFB; *P < 0.05 compared to NFB; ^#^ *P* < 0.05 compared to NFBD; ^$^ *P* < 0.05 compared to EFB; ^@^ *P* < 0.05 compared to EFBD.

NFB: normal folic acid, normal vitamin B_12_; NFBD: normal folic acid, vitamin B_12_ deficient; EFB: excess folic acid, normal vitamin B_12_; EFBD: excess folic acid, vitamin B_12_ deficient; NFBDO: normal folic acid, vitamin B_12_ deficient, omega-3 supplemented EFBDO: excess folic acid, vitamin B_12_ deficient, omega-3 supplemented.

## References

[1] Yajnik CS, Deshmukh US (2008). Maternal nutrition, intrauterine programming and consequential risks in the offspring. *Reviews in Endocrine and Metabolic Disorders*.

[2] van Wijk N, Watkins CJ, Hageman RJJ (2012). Combined dietary folate, vitamin B-12, and vitamin B-6 intake influences plasma docosahexaenoic acid concentration in rats. *Nutrition and Metabolism*.

[3] Finkelstein JD (1998). The metabolism of homocysteine: pathways and regulation. *European Journal of Pediatrics*.

[4] Stead LM, Brosnan JT, Brosnan ME, Vance DE, Jacobs RL (2006). Is it time to reevaluate methyl balance in humans?. *American Journal of Clinical Nutrition*.

[5] da Costa K-A, Rai KS, Craciunescu CN (2010). Dietary docosahexaenoic acid supplementation modulates hippocampal development in the pemt-/- mouse. *Journal of Biological Chemistry*.

[6] Selley ML (2007). A metabolic link between S-adenosylhomocysteine and polyunsaturated fatty acid metabolism in Alzheimer’s disease. *Neurobiology of Aging*.

[7] Pynn CJ, Henderson NG, Clark H, Koster G, Bernhard W, Postle AD (2011). Specificity and rate of human and mouse liver and plasma phosphatidylcholine synthesis analyzed in vivo. *Journal of Lipid Research*.

[8] Watkins SM, Zhu X, Zeisel SH (2003). Phosphatidylethanolamine-N-methyltransferase activity and dietary choline regulate liver-plasma lipid flux and essential fatty acid metabolism in mice. *Journal of Nutrition*.

[9] Huang T, Wahlqvist ML, Li D (2010). Docosahexaenoic acid decreases plasma homocysteine via regulating enzyme activity and mRNA expression involved in methionine metabolism. *Nutrition*.

[10] Huang T, Wahlqvist ML, Li D (2012). Effect of n-3 polyunsaturated fatty acid on gene expression of the critical enzymes involved in homocysteine metabolism. *Nutrition Journal*.

[11] Refsum H (2001). Folate, vitamin B12 and homocysteine in relation to birth defects and pregnancy outcome. *British Journal of Nutrition*.

[12] Roy S, Kale A, Dangat K, Sable P, Kulkarni A, Joshi S (2012). Maternal micronutrients (folic acid and vitamin B12) and omega 3 fatty acids: implications for neurodevelopmental risk in the rat offspring. *Brain and Development*.

[13] Kulkarni A, Dangat K, Kale A, Sable P, Chavan-Gautam P, Joshi S (2011). Effects of altered maternal folic acid, vitamin B12 and docosahexaenoic acid on placental global DNA methylation patterns in wistar rats. *PLoS ONE*.

[14] Wadhwani NS, Manglekar RR, Dangat KD, Kulkarni AV, Joshi SR (2012). Effect of maternal micronutrients (folic acid, vitamin B12) and omega 3 fatty acids on liver fatty acid desaturases and transport proteins in Wistar rats. *Prostaglandins Leukotrienes and Essential Fatty Acids*.

[15] Sable P, Dangat K, Kale A, Joshi S (2011). Altered brain neurotrophins at birth: consequence of imbalance in maternal folic acid and vitamin B_12_ metabolism. *Neuroscience*.

[16] Wadhwani NS, Dangat KD, Joshi AA, Joshi SR (2013). Maternal micronutrients and omega 3 fatty acids affect placental fatty acid desaturases and transport proteins in Wistar rats. *Prostaglandins Leukotrienes and Essential Fatty Acids*.

[17] Sundrani DP, Reddy US, Joshi AA (2013). Differential placental methylation and expression of VEGF, FLT-1 and KDR genes in human term and preterm preeclampsia. *Clinical Epigenetics*.

[18] Folch J, Lees M, Sloane Stanley GH (1957). A simple method for the isolation and purification of total lipides from animal tissues. *The Journal of Biological Chemistry*.

[19] Percy P, Vilbergsson G, Percy A, Mansson J-E, Wennergren M, Svennerholm L (1991). The fatty acid composition of placenta in intrauterine growth retardation. *Biochimica et Biophysica Acta: Lipids and Lipid Metabolism*.

[20] Zhou X, Arthur G (1992). Improved procedures for the determination of lipid phosphorus by malachite green. *Journal of Lipid Research*.

[21] Rouser G, Fleischer S, Yamamoto A (1970). Two dimensional thin layer chromatographic separation of polar lipids and determination of phospholipids by phosphorus analysis of spots. *Lipids*.

[22] Koike T, Ishida G, Taniguchi M (1998). Decreased membrane fluidity and unsaturated fatty acids in Niemann-Pick disease type C fibroblasts. *Biochimica et Biophysica Acta: Molecular Basis of Disease*.

[23] Hauner H, Much D, Vollhardt C (2012). Effect of reducing the n-6:n-3 long-chain PUFA ratio during pregnancy and lactation on infant adipose tissue growth within the first year of life: an open-label randomized controlled trial. *American Journal of Clinical Nutrition*.

[24] Simopoulos AP (1991). Omega-3 fatty acids in health and disease and in growth and development. *American Journal of Clinical Nutrition*.

[25] Dwarkanath P, Muthayya S, Thomas T (2009). Polyunsaturated fatty acid consumption and concentration among South Indian women during pregnancy. *Asia Pacific Journal of Clinical Nutrition*.

[26] McCaddon A, Hudson PR (2007). Methylation and phosphorylation: a tangled relationship?. *Clinical Chemistry*.

[27] Mikael LG, Pancer J, Jiang X, Wu Q, Caudill M, Rozen R (2013). Low dietary folate and methylenetetrahydrofolate reductase deficiency may lead to pregnancy complications through modulation of ApoAI and IFN-*γ* in spleen and placenta, and through reduction of methylation potential. *Molecular Nutrition and Food Research*.

[28] Watkins D, Rosenblatt DS (2012). Update and new concepts in vitamin responsive disorders of folate transport and metabolism. *Journal of Inherited Metabolic Disease*.

[29] Resseguie M, Song J, Niculescu MD, da Costa K-A, Randall TA, Zeisel SH (2007). Phosphatidylethanolamine N-methyltransferase (PEMT) gene expression is induced by estrogen in human and mouse primary hepatocytes. *FASEB Journal*.

[30] Vance DE, Walkey CJ, Cui Z (1997). Phosphatidylethanolamine N-methyltransferase from liver. *Biochimica et Biophysica Acta: Lipids and Lipid Metabolism*.

[31] Zhu X, Zeisel SH (2005). Gene expression profiling in phosphatidylethanolamine N-methyltransferase knockout mice. *Molecular Brain Research*.

[32] Sharma NK, Langberg KA, Mondal AK, Das SK (2013). Phospholipid biosynthesis genes and susceptibility to obesity: analysis of expression and polymorphisms. *PLoS ONE*.

[33] Phillips T (2008). The role of methylation in gene expression. *Nature Education*.

